# Assessing Bias in Population Size Estimates Among Hidden Populations When Using the Service Multiplier Method Combined With Respondent-Driven Sampling Surveys: Survey Study

**DOI:** 10.2196/15044

**Published:** 2020-06-15

**Authors:** Sungai T Chabata, Elizabeth Fearon, Emily L Webb, Helen A Weiss, James R Hargreaves, Frances M Cowan

**Affiliations:** 1 Centre for Sexual Health and HIV/AIDS Research Harare Zimbabwe; 2 Department of Global Health and Development London School of Hygiene and Tropical Medicine London United Kingdom; 3 UK Medical Research Council Tropical Epidemiology Group London School of Hygiene and Tropical Medicine London United Kingdom; 4 Department of Public Health, Environments and Society London School of Hygiene and Tropical Medicine London United Kingdom; 5 Department of International Public Health Liverpool School of Tropical Medicine Liverpool United Kingdom

**Keywords:** service multiplier method, respondent-driven sampling, population size estimation, female sex workers, key populations, HIV, Zimbabwe

## Abstract

**Background:**

Population size estimates (PSEs) for hidden populations at increased risk of HIV, including female sex workers (FSWs), are important to inform public health policy and resource allocation. The service multiplier method (SMM) is commonly used to estimate the sizes of hidden populations. We used this method to obtain PSEs for FSWs at 9 sites in Zimbabwe and explored methods for assessing potential biases that could arise in using this approach.

**Objective:**

This study aimed to guide the assessment of biases that arise when estimating the population sizes of hidden populations using the SMM combined with respondent-driven sampling (RDS) surveys.

**Methods:**

We conducted RDS surveys at 9 sites in late 2013, where the Sisters with a Voice program (the program), which collects program visit data of FSWs, was also present. Using the SMM, we obtained PSEs for FSWs at each site by dividing the number of FSWs who attended the program, based on program records, by the RDS-II weighted proportion of FSWs who reported attending this program in the previous 6 months in the RDS surveys. Both the RDS weighting and SMM make a number of assumptions, potentially leading to biases if the assumptions are not met. To test these assumptions, we used convergence and bottleneck plots to assess seed dependence of RDS-II proportion estimates, chi-square tests to assess if there was an association between the characteristics of FSWs and their knowledge of program existence, and logistic regression to compare the characteristics of FSWs attending the program with those recruited to RDS surveys.

**Results:**

The PSEs ranged from 194 (95% CI 62-325) to 805 (95% CI 456-1142) across 9 sites from May to November 2013. The 95% CIs for the majority of sites were wide. In some sites, the RDS-II proportion of women who reported program use in the RDS surveys may have been influenced by the characteristics of selected seeds, and we also observed bottlenecks in some sites. There was no evidence of association between characteristics of FSWs and knowledge of program existence, and in the majority of sites, there was no evidence that the characteristics of the populations differed between RDS and program data.

**Conclusions:**

We used a series of rigorous methods to explore potential biases in our PSEs. We were able to identify the biases and their potential direction, but we could not determine the ultimate direction of these biases in our PSEs. We have evidence that the PSEs in most sites may be biased and a suggestion that the bias is toward underestimation, and this should be considered if the PSEs are to be used. These tests for bias should be included when undertaking population size estimation using the SMM combined with RDS surveys.

## Introduction

### Background

In sub-Saharan Africa, female sex workers (FSWs) are at increased risk of HIV acquisition compared with the general population [1,2]. The Joint United Nations Programme on HIV/AIDS recommends targeted HIV surveillance among FSWs and other highly at-risk yet socially marginalized populations [3]. Population size estimates (PSEs) of these key populations are important for the design and evaluation of public health policy and serve as the basis for allocation of resources for treatment and prevention programs as well as informing modeled estimates of the epidemic [4]. However, there is no *gold standard* population size estimation method; estimates are subject to a range of different biases, and studies employing multiple approaches can show a wide variance in the estimates from each method [5-7]. Methods and standards for investigating and reporting assumptions and likely biases would improve the ability of policymakers to interpret and utilize PSEs appropriately.

The service multiplier method (SMM) is a commonly used method to estimate the size of key populations. The method uses 2 data sources [5-12], one of which is a count or listing of clients who are accessing a service, for example, the number of FSWs who attended a certain program or who were arrested by the police over a given period. The second data source is a probability-based sample of the population [3,11,13] in which participants are asked about their attendance at that program or arrest over the same period. The service usage count is divided by the proportion of participants in the survey who report using the service within the given time frame to yield a PSE.

In recent applications, respondent-driven sampling (RDS) surveys have been used to obtain a probability-based estimate of the proportion of the target population who are service users [5,7,11]. RDS exploits the social network structure of hard-to-reach populations for recruitment. If a given set of assumptions holds, weighted data from RDS can be interpreted as providing a representative sample of the network of the population sampled [14,15]. Although RDS has become an increasingly popular means of surveying key populations, the extent to which RDS estimates can be taken as representative has been questioned [16-18]. Investigating the sampling process over the network against assumptions can help us understand potential biases. There are now guidelines for conducting relevant diagnostics [19] and reporting them [20], but there is a need to illustrate the use of this guidance for use in obtaining PSEs with the SMM.

In addition to the SMM, various approaches for population size estimation have been used, including the enumeration method [3,12], the census method [3], the capture recapture method [3,12,21,22], and the unique object multiplier method [3,23]. As recommended, triangulating data from multiple methods have also been used to estimate the size of hard-to-reach populations [5,7,10]. In some settings, a high degree of agreement between methods has been found [12], whereas in other settings, there was evidence of bias between methods that could go in either direction [24,25].

### Objectives

In this paper, we build on existing guidance for implementing the SMM with RDS data [11] to critically appraise the assumptions and likely biases arising from using the SMM and RDS surveys to estimate the population sizes of FSWs at 9 sites in Zimbabwe, providing an illustrative example for assessing bias in future applications of the method.

## Methods

We first describe the data sources used, our application of the SMM, and then our approach to investigating the degree to which our study met the methodological assumptions and the potential resulting biases.

### Data Sources

Service data come from the *Sisters with a Voice* program (hereafter, *the program*) run on behalf of Zimbabwe’s National AIDS Council and Ministry of Health and Child Care. The program provides reproductive and sexual health services to women, identifying themselves as sex workers [26]. During their first visit to the program, FSWs are given a unique program identifier so that their visits to the program can be linked over geography and time [26]. For each individual who attends a program site, her unique identifier, date of visit, demographic information, HIV testing history, and the main reason for the visit are recorded. The program identifier is a combination of the first 2 letters of the name of the site where they first accessed program services and some numbers. The identifier should not be missing because it is a requirement for a woman to access services and in the event that they have forgotten their identifier, demographics are used to retrieve their history as well their identifier.

The probability-based sample comes from a baseline RDS survey of the Sisters Antiretroviral therapy Program for Prevention of HIV—an Integrated Response (SAPPH-IRe) trial, a cluster randomized controlled trial that was conducted among FSWs at 14 different sites across Zimbabwe in November and December 2013 (PACTR201312000722390) [27,28]. RDS recruitment took a maximum of 35 days across the 14 sites. In this PSE study, we included 9 sites that had had the program operational for at least six months before the baseline survey. These were all small towns and truck stops, not big cities. The estimated population size of all adult females aged 15 to 49 years during the 2012 census at these 9 sites was 33,302 at site 1, 8399 at site 2, 8694 at site 3, 15,407 at site 4, 10,329 at site 5, 7484 at site 6, 26,745 at site 7, 9085 at site 8, and 30,633 at site 9 [29]. Women were eligible to participate in the SAPPH-IRe baseline trial survey if they were aged ≥18 years on the survey date; had exchanged vaginal or anal sex for money, goods, or gifts at one of the study sites in the past month; and presented a valid recruitment coupon as explained below [30]. We asked survey participants for information on sociodemographics, sexual behavior, and HIV testing practices.

To initiate RDS recruitment, we purposively sampled 6 to 8 participants (*seeds*) from subgroups of the target population at each site, through the mapping of sex work in each community by geography, age, and sex work typology [31,32]. Seeds were not identified through program attendance to avoid bias. After participation in the survey, participants who were seeds were each provided with 2 uniquely coded coupons to recruit their peers [15,30,33]. Recruited peers then undertook study procedures and were further provided with 2 coupons that they used to recruit more members of the target population [14,15,19]. The process proceeded until the desired sample size (determined according to the trial’s primary outcome [31]) was attained, with 5 waves of recruitment following seeds, to approximately 200 FSWs at each site.

### Determining Unique Visits to the Program

To determine *M*, the number of visits to the program of unique women within the reference period, FSWs were counted only once using their identifier [11]. We excluded women aged <18 years to match the eligibility criteria for RDS participation, which was ≥18 years. We did not make any other restrictions as the RDS was attempting to sample from the same group of women accessing the program. Visits to the program by unique FSWs at each site were assumed to have happened at a constant rate, therefore following a Poisson distribution with the mean number of counts being the number of FSWs who were counted to have attended the program in the specified 6 months [11]. We used the normal approximation to Poisson distribution with the mean and variance equal to the number of FSWs who attended the program to determine the variability in the number of FSWs who attended the program at each site in the specified 6 months [11].

### Population Size Estimation

We applied the formula for the SMM, 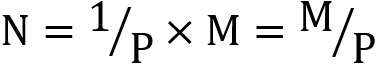 where *N* is the estimated population size of FSWs at each site, *P* is the RDS-adjusted population proportion of FSWs who reported program attendance 6 months before the RDS survey, and *M* is the total number of FSWs who attended the program within a period of 6 months before the RDS survey [5,7,11]. The proportion of women who reported attending the program in the previous 6 months was determined by first asking if the participant had heard of the program and then asking if they had attended in this time. To solicit for the last 6-month recall period for program attendance, the question in the RDS questionnaire relating to this was, “In the past 6 months, i.e. since dd/mm/yyyy, have you attended the *Sisters with a Voice* clinic.”

The RDS-II estimator was used to estimate P [34], and the network size used for weighting was the number of FSWs a participant would consider recruiting to the study among the total number of FSWs they knew would meet the eligibility criteria, and whom they had met in the last month. The network size question was asked after 2 follow-up questions and in the following order: How many sex workers do you know personally who live in your area, who are over 18, where you know their name and they know yours?; How many of those sex workers who you know personally have you seen in the last month?; and How many of those sex workers who you know personally would you consider recruiting to the study?

As recommended, we used the delta method to estimate the variance of *N* by combining the variances of *P* and *M* using the following formula: 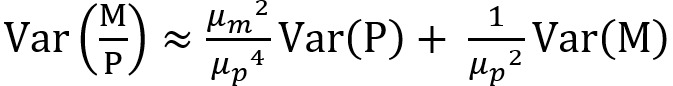 where *μ_m_* is the mean of *M* and *μ_p_* is the mean of P [11,35].

### Checking the Validity of Population Size Estimates

The SMM makes at least four assumptions, including (1) all members of the population being counted should have a chance of being included in both sources [3,11], (2) data sources should have the same and clear time references, age ranges, geographic areas, and individuals should not be counted more than once in each data source [3,7,11], (3) the 2 data sources should be independent of each other, that is, the inclusion of individuals in one source should not be related to the inclusion of individuals in the other source [3,11], and (4) the representative data source should be a random sample of the target population [7,11]. In our case, this latter assumption relates to the extent to which the (weighted) RDS survey sample can be treated as a representative sample, that is, met the assumptions of the RDS estimation.

For RDS-II estimates to be considered unbiased, assumptions including reciprocity, sampling with replacement, a completely connected networked population at each site, accurate report of personal network size, final sample independent of the original seeds, and random recruitment have to be satisfied [14,19,33,34,36-40]. We used existing guidance relating to RDS-II diagnostics [19] and interpreted them for their effect on the PSEs.

Reciprocity is an assumption of the Markov process, which states that if individual A recruited individual B, then in principle, B could have recruited A [36]. Given the dual system of incentives, this assumption is most likely to hold because participants would prefer to pass coupons to their friends and acquaintances rather than strangers [38]. The assumption is violated if respondents recruit strangers [36]. Sampling with replacement is also a Markov assumption that states that the respondent could be contacted again to participate in a study more than once [14,33,36]. Sampling with replacement assumption is violated when using RDS-I or RDS-II estimators, because in real-life RDS studies, sampling is without replacement, that is, the same individual cannot participate more than once in the survey. One could choose to use the RDS successive sampling estimator, which does not rely on the sampling with replacement assumption [41], but this estimator requires a PSE to already be available. A completely networked population requires that individuals from the target population should know each other and should communicate [36]. If individuals do not know each other, then it is not possible to come up with a representative sample of the sampled population because some individuals will not be accessible through the network and hence have zero probability of inclusion. Accurate report of personal network size by each RDS survey participant is important because network size is used in the computation of weights [34]. The final sample that is independent of the original seeds is the RDS-II estimator assumption that the sampling waves are sufficiently large such that the final estimates are independent of the bias that can be induced by the purposively selected seeds [14,19]. Another assumption of the RDS-II estimator is random recruitment, which states that respondents recruit randomly from their personal network [33,36]. This assumption is violated if recruiters preferentially recruit recruitees with particular characteristics from among their personal networks [36].

Other potential biases in *P* include recall bias where women may misremember dates and/or may not have recognized a service they visited as the program service and mobility (including mobility in and out of sex work) as a sampling bias where women who access the program may not be sampled at the time of the survey, and those who are sampled may not have potentially used these services over the past 6 months. A bias in the estimation of *M* could arise if the program failed to perfectly identify unique women visiting in the reference period.

We, therefore, investigated some of the RDS and SMM assumptions listed in Table 1 that were possible to investigate using available data and considered the resulting potential for biases in the PSEs.

**Table 1:** Respondent-driven sampling and service multiplier method assumptions.

Assumption			Criteria	Expected outcome
**Representative data source should be a random sample of the target population**				
	**Check all RDS-II^a^ assumptions**			
		Reciprocity (N/A^b^)	Ask participants’ relationship to the person who gave them a study coupon and if they say *stranger* then reciprocity will not be fulfilled.	Participants more likely to be recruited by friends and acquaintances.
		Sampling with replacement (N/A)	Always violated in real-life RDS^c^ studies, when the RDS successive sampling estimator is not used.	—^d^
		Accurate report of personal network size (N/A)	Sensitivity analysis of different network size questions.	RDS estimates should agree with each other regardless of different network size questions used.
		Final sample independent of the original seeds	Assess whether seed dependence was removed using convergence plots.	Overall estimate of *P* converges to the final estimate of *P* and remains stable as additional participants are recruited.
		Completely connected networked population at each site	Assess whether the FSW^e^ population is networked using bottleneck plots.	Estimate of *P* from individual seeds converge to a shared estimate.
		Random recruitment	Assess whether there is an indication of nonrandom recruitment by measuring recruitment homophily.	Recruitment homophily should be approximately 1.
	Two data sources combined are drawn from the same population, with the RDS data being representative of the target population		Compare sociodemographic and other characteristics of RDS surveys participants reporting program attendance with records of program attenders for the same time reference using logistic regression.	No evidence of difference in characteristics of RDS surveys participants who report program attendance within the reference period and the characteristics of program attenders in the program dataset during the reference period.
All members of the population being counted should have a chance of being included in both sources			Assess if all RDS surveys participants are familiar with the existence of the program by using chi-square tests to compare characteristics of individuals who had ever heard of the program with those who had not across sites.	No evidence of difference between individuals who had ever heard of the program with those who had not.
Data sources should have the same and clear time references, age ranges, geographic areas and individuals should not be counted more than once in each data source.			Assess if time references, age ranges and geographic areas of RDS and program data are similar or not; deduplicate program data if participants visited the program several times during the reference period.	Report if time references, age ranges and geographic areas are similar or not. Deduplicated program data.
The 2 data sources should be independent of each other, that is inclusion of individuals in 1 source should not be related to the inclusion of individuals in the other source.			Do not identify seeds and participants in general through the program; given that seed participants might also be more likely to be program attenders, even if they are not selected on this basis, assess convergence of *P* over time for evidence of seed dependence using convergence plots.	Report how RDS participants were identified and recruited; overall estimate of *P* converges to the final estimate of *P* and remains stable as additional participants are recruited.

^a^RDS-II: RDS Volz-Heckathorn estimator.

^b^N/A: denotes the assumptions that could not be investigated with the data available in this study.

^c^RDS: respondent-driven sampling.

^d^Assumption always violated when other RDS estimators (not the RDS successive sampling estimator) are used.

^e^FSWs: female sex workers.

#### Assessing Whether Seed Dependence Was Removed

In the RDS framework, seeds are selected purposively with the assumption that if recruitment is done with a sufficiently large number of waves, then the final sample would be independent of the seed characteristics [14]. We used convergence plots to examine whether the cumulative estimate of *P* stabilizes as the sample size increases [19]. A convergence plot shows the estimate of the RDS proportion on the vertical axis and the cumulative RDS sample size on the horizontal axis and is used to show how the overall RDS estimate changes as the sample size increases from wave 0 [19]. If the cumulative estimate appears to be continuing to rise or fall at close of the study, this could imply that the estimate was still dependent on the initial seed characteristics and could overestimate or underestimate the PSE.

#### Assessing Whether the Female Sex Worker Population Is Networked

We assessed whether the RDS-II weighted cumulative estimates of *P* varied by seed using bottleneck plots. The vertical axis of the bottleneck plot shows the estimate of the RDS proportion and the horizontal axis shows the cumulative RDS sample size, and these are shown separately for each seed (rather than altogether as in a convergence plot). If the individual seed estimates are not all converging toward a shared estimate, it might imply that the population is not really well networked, there is strong segregation into subgroups or that recruitment has got stuck in one branch of the network (a *bottleneck*).

#### Assessing Whether There Is an Indication of Nonrandom Recruitment

The indication of nonrandom recruitment was investigated by measuring recruitment homophily on *P*. Recruitment homophily is the tendency for women to recruit others like themselves with respect to reporting program attendance. In this case, it is the ratio of the number of recruits that have the same program attendance status as their recruiter to the number, we would expect by chance. If recruitment homophily on *P* is approximately 1, then there is little evidence of recruitment homophily, whereas values larger than 1 indicate more homophily.

#### Assessing Whether All Members of the Population Have a Chance of Being Included in the Program Data

The SMM requires that all members of the target population have a nonzero probability of being included in both the RDS survey and the program data [3,9], indicating that the target population should be familiar with the existence of the program. If members of the population with certain characteristics seem not to know about the existence of the program, then in theory they might have zero probability of being included in the program data, which violates the stated assumption of the SMM. We used the chi-square test of the RDS-II weighted proportions to compare the characteristics of individuals who had ever heard of the program with those who had not across sites. We used logistic regression models (interaction test of characteristics of individuals and site) to assess whether the association between characteristics and program knowledge differed among sites. The logistic regression model we used for each particular sociodemographic characteristic was log (*Y_i_*) = β_0_ + β_1_X*Site where *Y* is knowledge of the existence of a program and *X* represents each individual characteristic.

#### Assessing Whether the Two Data Sources Combined Are Drawn from the Same Population, With the Respondent-Driven Sampling Data Being Representative of the Target Population

We also assessed the SMM assumption that the 2 data sources to be combined should be drawn from the same population, with the RDS data being representative of this population [3]. Under this assumption, those sampled by RDS who reported attending the program 6 months before the RDS survey was conducted should be representative of those who actually attended the program in the same period of time, that is, they should be similar with respect to sociodemographic and other characteristics. If the characteristics are different, it might suggest that the women included in the RDS survey are not a representative sample of the population, or that there is bias in reporting program attendance among those in the RDS survey. We pooled both data sources and used logistic regression with data source as the outcome to compare the characteristics of FSWs who reported program use in the RDS survey with the characteristics of those in the program data to determine if this was likely the same population. RDS data were RDS-II weighted and program data were not weighted. Again, the interaction test of characteristics of individuals and site was used to assess whether the comparison between RDS data and program data differed among sites.

### Statistical Analysis

Unweighted descriptive analyses of program data and RDS-II weighted descriptive analyses of RDS data as well as comparison of the 2 data sources were performed using Stata version 14.2 (StataCorp LLC), and all the other RDS diagnostics were performed using RDS Analyst version 0.5.1, which is based on the RDS package for R [42]. PSE calculations were undertaken for each site separately, as were assessments of convergence, bottlenecks, and homophily. When investigating the association between characteristics of those who had and had not heard about the program, and between characteristics of those who visited the program and those recruited to RDS surveys, we pooled the data across sites. We investigated whether the associations in questions differed by site using an interaction test, and present regression analyses adjusting for a fixed term for site. In pooled site analyses, we used a normalized weighting variable. Pooling of RDS data overcame potential problems with small sample sizes but was a violation of the RDS assumption of 1 complete network component [43].

## Results

We recruited a total of 1739 FSWs from 8 seeds at site 1 and 6 seeds from each of the other 8 sites. Of these seeds at each site, only 1 seed had attended the program at site 1, 3 at sites 7 and 9, 5 at sites 2, 3, 5, 6, and 8, and all 6 at site 4.

### Population Size Estimates

The PSEs and 95% CIs calculated using the SMM are shown in Table 2.

**Table 2:** Population size estimates of female sex workers and 95% CI.

Site	RDS^a^ sample size	Number of FSWs^b^ who attended the program within the last 6 months (M)	SE for M^c^	Percent^d^ reporting visit (*P*; 95% CI)	SE for P	Population size estimate	SE for the population size estimate^e^	95% CI	Percent of FSWs among all women aged 15 to 49 years
1	220	57	7.4	20.3 (11.6-29.1)	4.5	281	70.1	133-407	0.8
2	196	100	10.0	25.0 (15.3-34.7)	4.9	400	87.2	225-566	4.8
3	153	111	10.5	46.1 (35.1-57.1)	5.7	241	37.2	166-311	2.8
4	202	372	19.2	68.7 (60.8-76.5)	4	541	42.0	455-619	3.5
5	197	84	9.2	20.6 (5.4-35.8)	7.8	408	160.4	93-722	3.9
6	200	28	5.3	14.3 (5.6-22.4)	4.2	194	67.0	62-325	2.6
7	165	34	5.8	11.0 (7.2-14.8)	1.9	310	75.4	162-458	1.2
8	198	46	6.8	16.7 (7.4-26.1)	4.8	275	88.7	101-449	3.0
9	208	165	12.8	20.5 (12.4-28.7)	4.2	805	175.1	456-1142	2.6

^a^RDS: respondent-driven sampling.

^b^FSWs: female sex workers.

^c^Calculated using the normal approximation to Poisson distribution.

^d^RDS-II adjusted percentages.

^e^Calculated using the delta method.

The number of women who attended program sites in the previous 6 months before the survey ranged from 28 at a site where the program was relatively new to 372 at a site where the clinic had been established for 2 years. The proportion of FSWs reporting program attendance varied from 11% to 69%. The highest PSE was 805 FSWs (95% CI 456-1142) and the lowest was 194 FSWs (95% CI 62-325). The 95% CIs for the majority of sites were wide (Table 2).

### Convergence Plots of P

**Figure 1:**
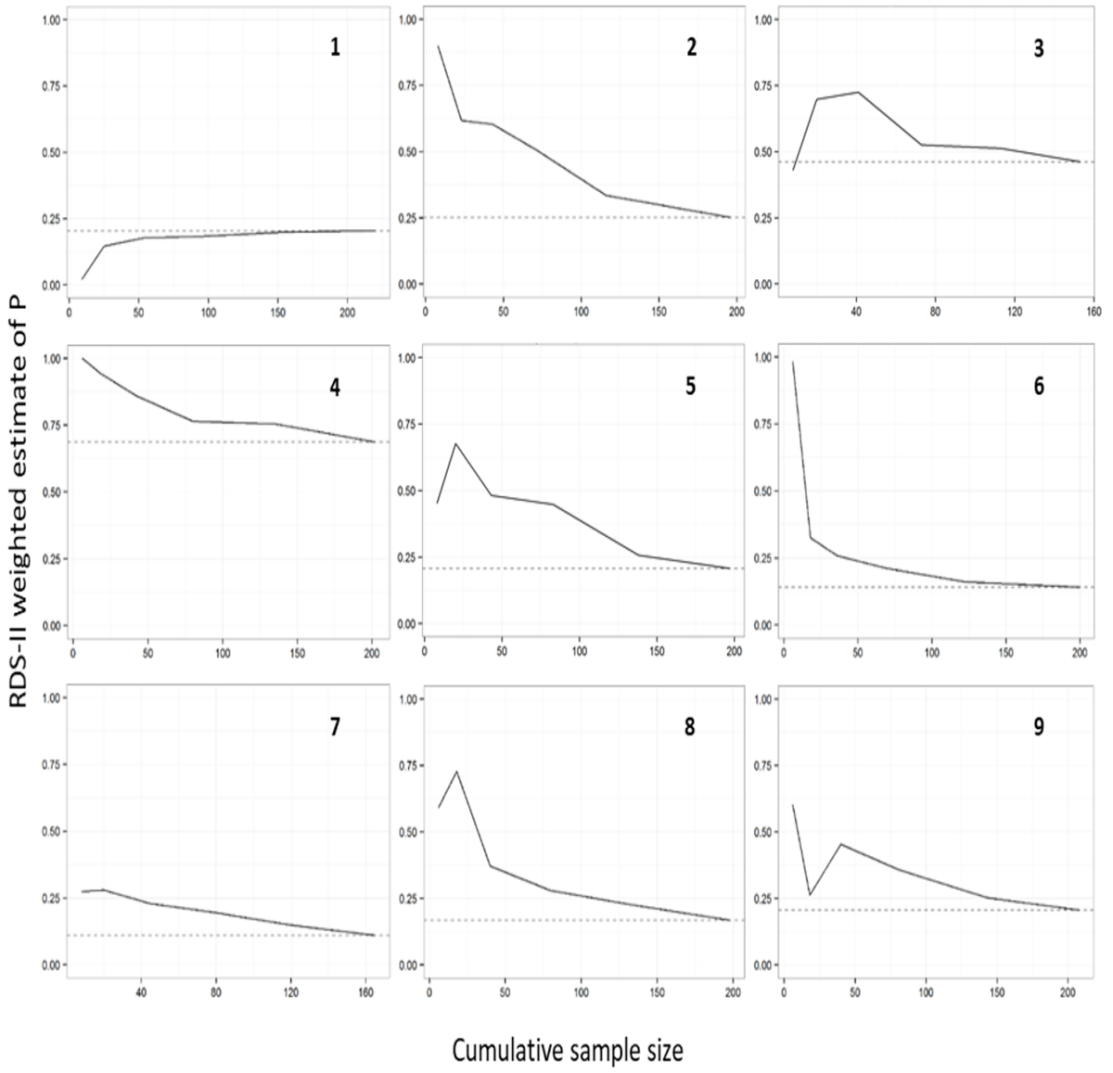
Site convergence plots. RDS-II: respondent-driven sampling Volz-Heckathorn estimator.

At sites 1 and 6, the estimate of *P* converged as the sample sizes increased, indicating that the final estimate of *P* might be independent of the seeds (Figure 1). However, at the other 7 sites, the estimate of *P* did not converge and continued to decline as recruitment continued, indicating that the final estimate was still influenced by the characteristics of the seeds and was likely an overestimate of *P*.

### Bottleneck Plots

**Figure 2:**
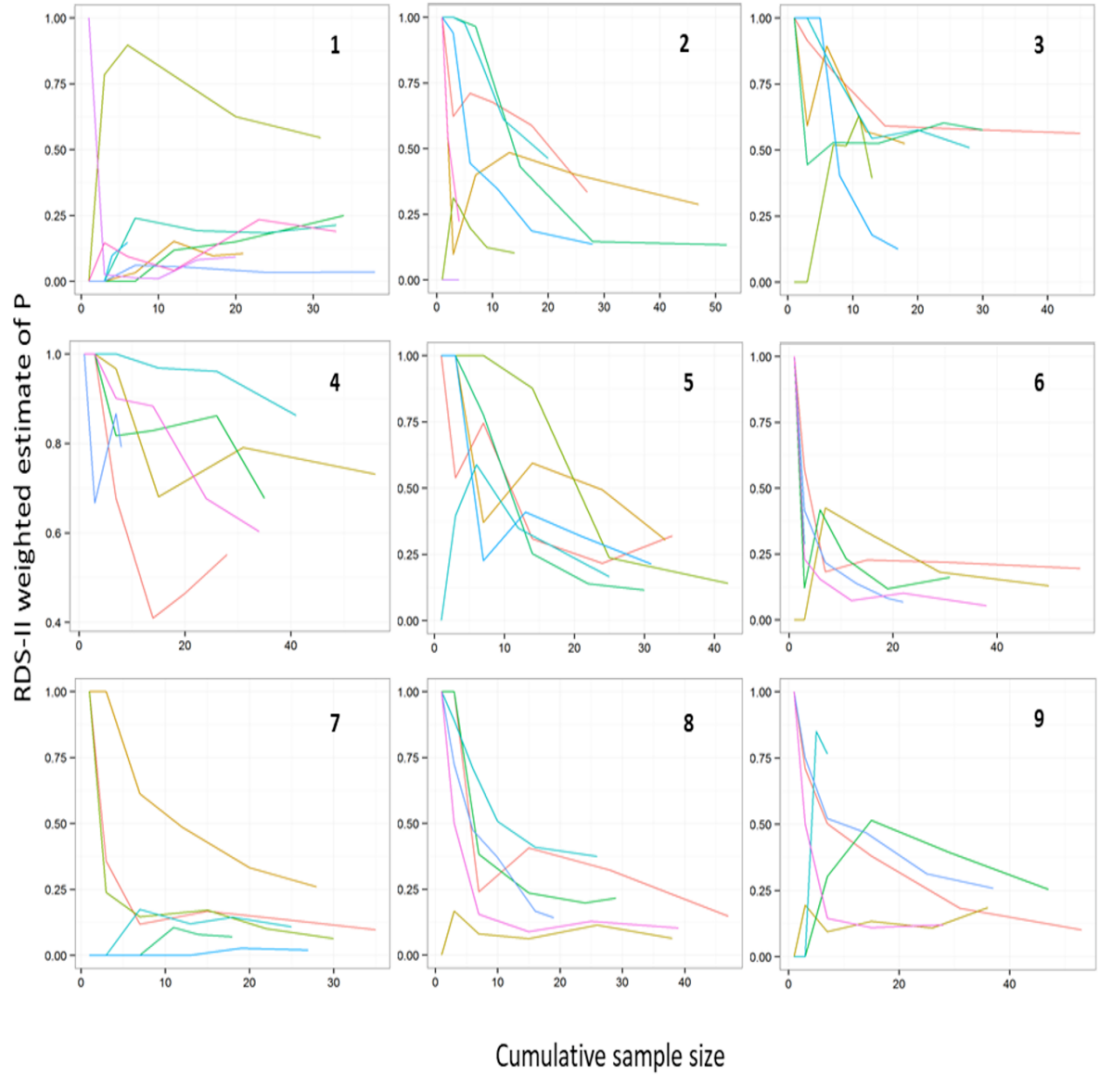
Site bottleneck plots. RDS-II: respondent-driven sampling Volz-Heckathorn estimator.

The bottleneck plots (Figure 2) at sites 5, 6, 7, and 8 show the individual tracks converging to a shared estimate, potentially indicating a lack of subgroups in the target population at these sites. The final estimates were 0.21 at site 5, 0.14 at site 6, 0.11 at site 7, and 0.17 at site 8. However, at sites 1, 2, 3, 4, and 9, where the final estimates were 0.20, 0.25, 0.46, 0.69, and 0.21, respectively, individual tracks did not converge, suggesting distinct subgroups.

### Recruitment Homophily

There was little evidence of recruitment homophily, ranging from 0.9 to 1.1 at sites 2 to 9, suggesting a weak tendency for women to recruit others like themselves with respect to reporting program attendance in the past 6 months. However, at site 1, recruitment homophily was moderate (1.4; Table 3).

**Table 3:** Recruitment homophily in P.

Site	Recruitment homophily in *P*
1	1.39
2	1.14
3	1.04
4	0.96
5	1.05
6	0.97
7	1.00
8	0.92
9	1.21

### Distribution of Respondent-Driven Sampling Survey Participants According to Their Knowledge of the Existence of a Program

There was little evidence of an association between the majority of sociodemographic characteristics and knowledge of program existence. Evidence of association was seen for education, where a higher proportion of women who reported secondary school or higher had heard about the program compared with those who reported primary school or none (44% vs 36%; *P*=.02), and for HIV testing, where relatively more women who had ever been tested for HIV had knowledge of program existence compared with those who had not tested (42% vs 27%; *P*=.01; Table 4). There was also little evidence that these relationships were different among sites for the majority of sociodemographic characteristics, except for the number of close friends (*P*=.02) and number of children aged under 18 years (*P*=.01).

**Table 4:** Association between sociodemographic characteristics and knowledge of program existence among respondent-driven sampling survey participants by site.

Characteristics			Total individuals (N=1739), n		Individuals who have ever heard about the program (N=803), n (%)		Comparison *P* value^a^		Interaction *P* value^b^
**Age (years)**						.40		.40	
	18-24	418		174 (36.8)					
	25-29	424		202 (40.1)					
	30-39	597		284 (43.1)					
	40+	299		143 (44.6)					
**Marital status**						.06		.10	
	Never married	356		170 (42.1)					
	Married or widowed	335		139 (33.3)					
	Divorced or separated	1047		494 (43.31)					
**Education**						.02		.47	
	Primary or none	531		209 (35.7)					
	Secondary or higher	1192		590 (44.13)					
**Age when started sex work (years)**						.87		.23	
	<18	343		157 (41.5)					
	18-24	630		284 (39.3)					
	25-29	398		195 (42.9)					
	>30	367		167 (41.2)					
**Duration at the site (years)**						.32		.52	
	0-1	186		86 (36.8)					
	2-5	587		245 (39.3)					
	>5	956		468 (43.7)					
**Number of FSWs^c^ who are close friends**						.13		.02	
	0	79		43 (49)					
	1	372		179 (40.6)					
	2-4	1031		457 (38.83)					
	>5	256		124 (50.4)					
**Number of commercial partners in last week**						.24		.32	
	0	132		59 (36.6)					
	1-4	705		312 (38.2)					
	5-9	415		205 (45.2)					
	>10	486		227 (44.7)					
**Number of children** **<** **18 years**						.24		.01	
	0	360		167 (37.5)					
	1-2	912		425 (43.8)					
	>3	466		211 (38.7)					
**Ever been tested for HIV**									
	No	110		36 (27.0)					
	Yes	1628		767 (42.02)					
**How many times been tested for HIV^d^**						.50		.89	
	1	292		124 (38.3)					
	2-4	910		431 (42.2)					
	>5	417		209 (44.9)					
**Most recent HIV test result^d^**						.36		.93	
	Negative	898		413 (40.7)					
	Positive	720		349 (44.0)					
**Condom use**						.91		.32	
	Consistent	1180		540 (40.79)					
	Nonconsistent	369		171 (40.3)					

^a^Chi-square *P* value for the association of each characteristic with knowledge of program existence.

^b^*P* value assessing the interaction between sociodemographic characteristics and site.

^c^FSWs: female sex workers.

^d^Among those ever tested for HIV.

### Comparison of Program Data With Respondent-Driven Sampling Data

There was little evidence of differences in the distribution of most sociodemographic characteristics between women who attended the program and those who reported program use in RDS data (Table 5). Evidence of a difference was only seen for duration at the site, where a higher proportion (84%) of women who reported program use in the RDS survey reported that they had lived at their respective sites for 2 or more years compared with 75% of those who actually attended the program. There was also no evidence that the distribution of these characteristics was different between sites.

**Table 5:** Comparison of sociodemographic characteristics of individuals who attended the program and individuals who reported program use in respondent-despondent sampling surveys.

Characteristic		Individuals who reported program use in RDS^a^ data (N=535), n (%^a^)	Individuals who actually attended the program (N=997), n (%)	Comparison *P* value^b^	Interaction *P* value^c^
**Age (years)**				.88	.67
	18-24	108 (22.4)	187 (19.2)		
	25-29	137 (22.7)	246 (25.2)		
	30-39	192 (35.1)	370 (38.0)		
	>40	98 (19.8)	171 (17.6)		
**Marital status**				.61	.52
	Never married	110 (19.4)	194 (19.8)		
	Married or widowed	93 (15.3)	192 (19.6)		
	Divorced or separated	332 (65.3)	594 (60.6)		
**Education**				.47	.16
	Primary or none	146 (31.7)	243 (28.0)		
	Secondary or higher	386 (68.3)	625 (72.0)		
**Duration at the site (years)**				.01	.22
	0-1	64 (16.1)	225 (25.3)		
	>2	467 (83.9)	666 (74.7)		
**Number of children under 18 years**				.42	.17
	0	108 (23.0)	238 (24.0)		
	1-2	288 (56.6)	593 (59.8)		
	>3	139 (20.4)	161 (16.2)		
**Ever been tested for HIV**				.18	.75
	No	26 (4.9)	64 (6.6)		
	Yes	509 (95.1)	911 (93.4)		
**Most recent HIV test result**				.42	.48
	Negative	262 (53.4)	442 (49.7)		
	Positive	242 (46.6)	447 (50.3)		

^a^RDS-II (respondent-driven sampling) weighted percentages.

^b^Wald *P* value comparing program data with RDS data.

^c^*P* value assessing the interaction between sociodemographic characteristics and the site.

## Discussion

### Principal Findings

We combined data on the proportion of FSWs recruited to RDS surveys in 9 Zimbabwean sites and who reported attending the program (*P*), with data relating to the program encounters at these same sites over the same recall period (M). Using these data, we estimated the size of the FSW population at each site using the SMM. Estimated population sizes ranged from 194 (95% CI 62-325) to 805 (95% CI 456-1142) across the sites for the period from June to December 2013, reflecting between 1% and 5% of the total female population aged 15 to 49 years in these sites.

We employed existing RDS diagnostics [19] alongside some additional analyses to explore potential biases affecting the PSEs. We found that FSWs who had accessed the program were more likely to be recruited earlier on in the RDS surveys. In the majority of sites, the estimate of program attendance, *P*, might have been overestimated, which would result in an underestimated PSE. The sources combined were likely not to be independent because some of our seed participants in the RDS surveys were program users who were more likely to recruit program users as evidenced by convergence and bottleneck plots. Having longer recruitment chains could have reduced our likelihood of getting stuck in a subgroup and allowed us to reach parts of the network not previously sampled. A positive correlation resulted in *P* being inflated, ultimately resulting in the underestimation of PSEs. This was also reported by Johnston et al [11] in their size estimation study. In the majority of sites, there was little evidence for high levels of recruitment homophily by program attendance (*P*), with the exception of 1 site. At this same site (site 1), although convergence had been achieved, the bottleneck plot appeared to show that program attendance might have differed substantially by the subnetwork of FSWs.

We found little evidence that women with particular characteristics were likely excluded from the program, suggesting that the SMM assumption that all members of the target population should have a nonzero probability of being included in both the RDS survey and the program was met. Characteristics of program attendees were similar to RDS participants, suggesting that the data sources were likely from the same population with the RDS surveys representative of the population, therefore partly satisfying the requirements of the SMM.

### Strengths and Limitations

The major strength of the SMM is that it can be implemented using data collected for other purposes [21,23,44], unlike other methods such as the enumeration method and the census method [3]. However, this can also be viewed as a weakness: if the existing data are poorly documented or are duplicated, the PSEs will be biased [11,13]. In most cases, sample size calculations for RDS surveys may not have been based on the need to estimate the population size with a reasonable level of precision [45], and the program might be poor in reaching a certain subset of the population of interest such that the subset will not be counted. Additionally, SMM is based on numerous assumptions, and the degree to which they are met is often not reported. The SMM can be expensive if RDS surveys are specifically conducted for population size estimation. On the other hand, this allows the collection of other data with the possibility of estimating population sizes using more than one method, for example, the RDS successive sampling size estimator [46] and a unique object multiplier [3].

This study has several strengths. Our simple and straightforward diagnostics were able to identify potential biases and suggest the potential direction of bias in the PSEs. The RDS survey data were carefully collected with an in-house coupon manager software to track coupons, verify them, and check that they were redeemed only once [32]. The definition of the population of interest was consistent across the program and RDS survey data [11]. Our program records allowed us to compare their characteristics with those recruited to RDS surveys. We clearly and consistently defined time references in both data sources, and this was a strength over other size estimation studies where inconsistent time references were reported [8,9]. Geographic areas in both data sources were clearly defined, and these were discrete urban or peri-urban settings such that anyone from around those specific areas could come to the program or participate in the RDS survey. Our size estimates for each site are plausible given other literature of the estimated proportion of adult women engaged in sex work in a setting similar to ours [47].

Study limitations include the inability to investigate all assumptions made by RDS and SMM. The SAPPH-IRe trial baseline was not set up to be used to estimate PSEs, and as such could not investigate all assumptions made by RDS and SMM. We were not able to assess the RDS assumption of accurate reporting of personal network size by participants. We also could not assess the SMM assumption that the 2 data sources should be independent of each other. We do not have data about every sex worker that a woman knows and all of their characteristics to assess whether the ones she recruits for the survey are a random sample or not (though this would be challenging to collect in practice). The assessment of convergence and bottleneck plots is rather qualitative and relies upon visually assessing graphics, which might result in making subjective conclusions.

Although we checked the design effect for the primary outcome of the trial for which these data were collected, which confirmed that the target sample sizes of 200 were adequate, we did not check the design effect for *P*, and we are not sure about the implications of this. To get an indication of whether the population of FSWs recruited to RDS surveys and those recruited to the program differed, and to assess whether women who had heard of the program differed to those who had not, we combined the RDS samples. This overcame the difficulty of making these assessments with small sample sizes, but it violates the RDS assumption of a completely networked population to do this [43].

### Recommendations

Although there is guidance on RDS sample size calculations [45,48] and guidance about assessing the assumptions made for RDS surveys [19], our findings indicate the importance of using RDS diagnostics to assess the estimate of *P* obtained for use in the multiplier method PSEs, and in assessing further multiplier method assumptions where data sources allow. We recommend that they are included when undertaking population size estimation using SMM combined with RDS surveys. PSEs are increasingly being taken up in policy making and by funders to set program targets, even at subnational levels. If the PSEs are assumed to be unbiased, programs may either be expected to reach people who do not exist or be inadequately funded to meet the needs of key populations who are undercounted.

We used a single multiplier for illustrative purposes, but in line with other groups, we recommend the use of more than one as multipliers are prone to unmeasurable bias [49]. PSEs may be considered unbiased when convergence has been reached, no bottlenecks, low homophily, program data are deduplicated, and the 2 study populations have similar characteristics among other criteria.

When incorporating the SMM in RDS surveys for population size estimation, it is important to understand the context in each site, which can be achieved through detailed mapping [5]. Key dynamics include seasonal migration patterns of the population of interest [50] to avoid overestimation in areas where they are immigrating and underestimation in areas where they are emigrating. The way that women meeting study criteria as a *sex worker* actually self-identify and are identified by their peers [51], as well as transition into and out of sex work, are important factors to consider. High-quality survey data are critical. It is recommended to include a large number of waves in RDS studies, although in some of our sites the overall population size was likely relatively small, practically limiting the number of waves that could be implemented. This might have been overcome by having fewer seeds, provided the full diversity of the FSW population could still be reached. There is a need to keep track of estimates based on program use by using convergence and bottleneck plots such that the sample size could possibly be increased if the estimates do not stabilize [19]. Some further areas of interest include data on reciprocity and questions to assess the random recruitment of the composition of personal networks (though this can be difficult to do in practice) to the RDS questionnaire to enable the further investigation of RDS assumptions [19].

Double counting of participants in program data needs to be minimized, as this could potentially result in overestimation of the PSEs. The program to be used in population size estimation should be accessible to all members of the target population, and members need to be given unique identifiers coupled with collection of additional information such that if they forget their program unique identifiers, they can easily be reminded. This will reduce the problem of duplication in the counting of individuals who attend the program on several occasions and partly contribute to the accurate calculation of PSEs. When estimating key population sizes, the SMM will ideally be triangulated with other population size estimation methods (capture-recapture, census, network scale-up, and SS-PSE). The size estimates obtained from each of these methods can be quite variable [5,7] such that results can be compared and more robust estimates such as the median of all the estimates can be used, with the lowest and highest estimates among the methods treated as the lower and upper confidence bounds, respectively [7].

### Conclusions

The SMM can be used to incorporate RDS proportion estimates [11]. Without a *gold standard* method for estimating the population sizes of hard-to-reach populations, the SMM is a recommended method to use [3,7]. We implemented a range of established and bespoke diagnostics in our application and suggest that it is important for researchers to use and publish similar diagnostics when using the SMM combined with RDS surveys.

## Abbreviations

FSW: female sex worker
PSE: population size estimate
RDS: respondent-driven sampling
SAPPH-IRe: Sisters Antiretroviral therapy Program for Prevention of HIV—an Integrated Response
SMM: service multiplier method
